# Fatores associados à soroprevalência de infecção por *Toxoplasma gondii* em gestantes: estudo transversal, Piraquara, 2024

**DOI:** 10.1590/S2237-96222026v35e20250708.pt

**Published:** 2026-10-05

**Authors:** Fernanda Paula da Silva Torres, Nicole Muniz Ferreira Gonçalves, Vivien Midori Morikawa, Yanna Dantas Rattmann, Denise Siqueira de Carvalho, Marlene da Cruz Magalhães Buffon

**Affiliations:** 1 Universidade Federal do Paraná, Programa de Pós-Graduação em Saúde Coletiva, Curitiba, PR, Brasil; 2 Universidade Federal do Paraná, Programa de Residência Multiprofissional em Saúde da Família, Curitiba, PR, Brasil; 3 Universidade Federal do Paraná, Departamento de Saúde Coletiva, Curitiba, PR, Brasil

**Keywords:** Estudos Soroepidemiológicos, Gestantes, Toxoplasmose, Toxoplasma gondii, Estudos Transversais, Estudios Seroepidemiológicos, Mujeres Embarazadas, Toxoplasmosis, Toxoplasma gondii, Estudios Transversales

## Abstract

**Objetivos:**

Estimar a soroprevalência de infecção por *Toxoplasma gondii* em gestantes da região do Guarituba, no município de Piraquara, Paraná, e analisar fatores sociodemográficos, clínicos e epidemiológicos associados à soropositividade.

**Métodos:**

Estudo transversal conduzido entre março e setembro de 2024 em quatro unidades básicas de saúde (UBS). As informações foram obtidas por entrevistas estruturadas e registros de pré-natal. Realizaram-se análise descritiva e regressão logística múltipla para estimar razões de chances (odds ratio, OR) e intervalos de confiança de 95% (IC95%).

**Resultados:**

Foram incluídas 240 gestantes. A soroprevalência de anticorpos IgG anti-T. gondii foi de 43,3% (IC95% 37,2; 49,7). Maiores chances de soropositividade foram observadas em gestantes de raça/cor da pele preta (OR 2,99; IC95% 1,07; 8,35), com quatro ou mais gestações (OR 2,04; IC95% 1,17; 4,14) e que consumiam peixe proveniente de fontes alternativas ao comércio formal (OR 1,62; IC95% 1,19; 2,20). Renda mensal ≥1 salário mínimo (OR 0,77; IC95% 0,62; 0,95) e o hábito de tratar a água destinada ao consumo (OR 0,42; IC95% 0,21; 0,81) apresentaram associação inversa.

**Conclusão:**

A soropositividade esteve associada a fatores sociodemográficos e alimentares, enquanto melhores condições socioeconômicas e o tratamento da água destinada ao consumo apresentaram associação inversa.

## Resumen

**Objetivo:**

Estimar la seroprevalencia de la infección por *Toxoplasma gondii* en mujeres embarazadas de la región de Guarituba, en el municipio de Piraquara, Paraná, Brasil, y analizar los factores sociodemográficos, clínicos y epidemiológicos asociados a la seropositividad.

**Métodos:**

Se realizó un estudio transversal entre marzo y septiembre de 2024 en cuatro unidades de atención primaria de salud. Los datos se obtuvieron mediante entrevistas estructuradas y registros prenatales. Se realizó un análisis descriptivo y una regresión logística múltiple para estimar las razones de probabilidades (*odds ratio*, OR) y los intervalos de confianza del 95% (IC95%).

**Resultados:**

Se incluyeron 240 mujeres embarazadas. La seroprevalencia de anticuerpos IgG anti-*T. gondii* fue del 43,3% (IC95%: 37,2; 49,7). Se observaron mayores probabilidades de seropositividad en mujeres embarazadas de raza/color negro (OR 2,99; IC95%: 1,07; 8,35), mujeres con cuatro o más embarazos (OR 2,04; IC95%: 1,17; 4,14) y que consumían pescado de fuentes distintas al comercio formal (OR 1,62; IC95%: 1,19; 2,20). Los ingresos mensuales ≥1 salario mínimo (OR 0,77; IC95%: 0,62; 0,95) y el hábito de tratar el agua potable (OR 0,42; IC95%: 0,21; 0,81) mostraron asociación inversa.

**Conclusión:**

La seropositividad se asoció con factores sociodemográficos y dietéticos, mientras que mejores condiciones socioeconómicas y el tratamiento del agua potable mostraron asociación inversa.

## Introdução

A toxoplasmose, infecção causada por *Toxoplasma gondii*, é uma zoonose com ampla distribuição global, com importantes implicações para a saúde pública, especialmente em países de baixa e média renda [1]. Estima-se que cerca de um terço da população mundial apresente soropositividade para *T. gondii*, com prevalências mais elevadas em regiões da América Latina e da América do Sul [2]. A infecção primária, bem como reativações em gestantes imunossuprimidas ou reinfecções durante a gestação, pode resultar em toxoplasmose congênita, associada a desfechos graves como aborto, microcefalia, retinocoroidite e calcificações cerebrais [3,4].

Classificada como doença de transmissão hídrica e alimentar, pode ocorrer pela ingestão de oocistos presentes em água ou alimentos contaminados com oocistos esporulados do parasito, eliminados no ambiente por fezes de felinos infectados, ou ainda pelo consumo de carnes cruas ou malcozidas contendo cistos teciduais [4]. 

No Brasil, a notificação da toxoplasmose gestacional tornou-se compulsória em 2016, e os registros têm aumentado progressivamente. Entre 2019 e 2023, as médias nacionais foram de 33,0 casos por 10 mil gestantes para toxoplasmose gestacional e 9,1 casos por 10 mil nascidos vivos para a forma congênita [5].

A avaliação da soroprevalência de anticorpos anti-*T. gondii* em gestantes permite caracterizar a proporção de mulheres previamente expostas ao parasito e a proporção de mulheres suscetíveis à infecção. Essa informação é relevante para a vigilância epidemiológica, pois auxilia na compreensão do potencial de ocorrência de infecção primária durante a gestação e no planejamento de ações de prevenção e acompanhamento no pré-natal.

Piraquara é um dos 29 municípios da Região Metropolitana de Curitiba, Paraná. Nesse município, entre 2019 e 2023, a média de casos de toxoplasmose gestacional foi de 104,2 por 10 mil gestantes, e a de toxoplasmose congênita, de 29,4 por 10 mil nascidos vivos. Ambos os indicadores foram superiores às médias nacionais [5,6]. No município, a região do Guarituba concentrou 67% dos casos notificados em 2023, evidenciando a distribuição desigual da doença [7]. Em 2024, a região contava com cerca de 51 mil habitantes, o que correspondia a 43% da população total de Piraquara. Reconhecido como região administrativa desde 2016, o Guarituba apresenta vulnerabilidade urbana e ambiental, marcada por ocupações irregulares e deficiências no saneamento básico [6,8]. Tal cenário posiciona a região como prioritária para ações de saúde pública. Investigar o perfil sorológico das gestantes da região torna-se relevante para compreender a proporção de mulheres previamente expostas e suscetíveis à infecção.

Sob a ótica da Saúde Coletiva, compreender as desigualdades sociais e territoriais é essencial para o enfrentamento de agravos evitáveis como a toxoplasmose. A abordagem “Uma Só Saúde” complementa essa perspectiva ao integrar dimensões humanas, animais e ambientais, evidenciando a interdependência entre condições de vida, ambiente e saúde no controle de zoonoses. Este estudo teve como objetivo estimar a soroprevalência de infecção por *T. gondii* em gestantes da região do Guarituba e analisar os fatores sociodemográficos, clínicos e epidemiológicos associados à soropositividade.

## Métodos

### Delineamento do estudo e contexto

Estudo transversal realizado entre março e setembro de 2024 na região do Guarituba, município de Piraquara, Paraná, Brasil. Em 2024, a região concentrava aproximadamente 51 mil habitantes, correspondendo a 42,9% da população municipal. A área é reconhecida por ser a maior ocupação irregular do estado e pela elevada vulnerabilidade social.

O estudo ocorreu nas quatro unidades básicas de saúde (UBS) da Regional Guarituba. A amostragem estratificada proporcional utilizada considerou o número médio de gestantes em acompanhamento pré-natal em cada unidade: 135 na UBS Carlos Jess; 86 na Maria Francelina dos Santos; 73 na Elfride Miguel de Oliveira; e 58 na Wanda Mallmann. 

No contexto do pré-natal na atenção primária à saúde, a investigação sorológica para toxoplasmose integra a rotina de exames gestacionais, conforme recomendações do Ministério da Saúde [9]. A sorologia para *Toxoplasma gondii* é solicitada no início do acompanhamento gestacional e, em gestantes suscetíveis, deve ser repetida a cada trimestre da gestação.

### Participantes

Foram incluídas no estudo gestantes de qualquer idade, residentes na área de abrangência das UBS da região do Guarituba, que estivessem em acompanhamento pré-natal em alguma das unidades durante o período de coleta e que tivessem realizado ao menos uma rotina de exames do pré-natal com registro de sorologia para anticorpos anti-*Toxoplasma gondii.*

As gestantes foram recrutadas independentemente do tempo de residência na área.

Foram excluídas gestantes menores de 18 anos desacompanhadas de responsável legal, gestantes sem caderneta de pré-natal ou sem registro de sorologia par*a T. gondii*, bem como aquelas que se recusaram a participar do estudo. 

As participantes presentes nas UBS foram convidadas após esclarecimentos sobre a pesquisa e incluídas por amostragem consecutiva, conforme comparecimento às unidades durante o período do estudo e aceite voluntário.

### Variáveis

O desfecho principal do estudo foi a soropositividade para anticorpos IgG anti-*Toxoplasma gondii*, classificada como reagente ou não reagente conforme registro na caderneta de pré-natal. A presença de IgG reagente foi considerada indicativa de exposição prévia ao parasito. As variáveis laboratoriais IgM e avidez de IgG, quando disponíveis, foram utilizadas apenas para caracterização sorológica das participantes e identificação de possíveis infecções recentes ou anteriores à gestação. As variáveis analisadas foram agrupadas em quatro domínios:

a) faixa etária (em anos: <25; 25-34; ≥35), estado civil, raça/cor da pele, escolaridade (anos de estudo: 1-8; 9-11; ≥12), situação ocupacional, fonte de renda, renda mensal da gestante (sem renda; <1; ≥1 salário mínimo), tempo de residência na área (em anos: ≤5; >5-10; >10) e características domiciliares e de saneamento;
b) Obstétricas de saúde: idade gestacional no momento da entrevista, trimestre de início do pré-natal, número de consultas, número de gestações (1; 2; 3; ≥4); tipo de serviço de acompanhamento de pré-natal e histórico de abortamentos;
c) Convívio com gatos: presença de felinos no domicílio, tipo de alimentação dos animais, manejo de fezes e acesso dos animais ao ambiente externo;
d) Práticas higiênico-alimentares: consumo de produtos de origem animal, consumo de carne crua ou malcozida, higienização de hortifrútis, fonte de água para consumo e realização de refeições fora do domicílio.

### Fontes de dados e mensuração

As informações foram obtidas por meio de entrevistas presenciais individuais realizadas em salas reservadas das UBS, utilizando-se questionário estruturado baseado na literatura sobre toxoplasmose gestacional, fatores de risco, hábitos de vida e condições socioambientais. O instrumento continha 39 questões referentes a características sociodemográficas, práticas alimentares e de higiene, histórico obstétrico e convívio com animais. 

Os resultados sorológicos (IgM, IgG e avidez de IgG para *T. gondii*), classificados como reagente ou não reagente, foram obtidos a partir dos registros na caderneta de pré-natal das gestantes, conforme anotação do médico responsável pelo pré-natal. Os exames foram realizados de acordo com rotina do pré-natal da rede municipal de saúde, por laboratório vinculado ao sistema público, utilizando metodologia padronizada pelo serviço. O teste de avidez de IgG, quando registrado, foi utilizado para interpretação dos casos com IgM e IgG reagentes. Alta avidez foi interpretada como infecção anterior à gestação, conforme preconiza o Ministério da Saúde [9]. 

O questionário foi previamente testado em estudo piloto com gestantes da mesma população, não incluídas na amostra principal, para avaliar clareza, adequação da linguagem e tempo de aplicação. As adaptações decorrentes do teste piloto foram incorporadas ao instrumento final. As entrevistas e a coleta de informações seguiram o mesmo procedimento em todas as UBS.

### Viés

Para reduzir vieses de informação, as entrevistas foram conduzidas individualmente em salas reservadas das UBS, com linguagem adaptada ao nível de compreensão das participantes e aplicação de questionário previamente testado em estudo piloto. As informações laboratoriais foram obtidas a partir dos registros na caderneta de pré-natal, conforme anotação do médico responsável pelo acompanhamento, utilizando dados gerados na rotina assistencial do pré-natal. Considera-se a possibilidade de viés de memória e desejabilidade social em variáveis autorreferidas relacionadas a hábitos alimentares e de higiene.

Para minimizar viés de seleção, foram incluídas gestantes elegíveis em acompanhamento pré-natal nas quatro UBS da Regional Guarituba durante o período de coleta, recrutadas por amostragem consecutiva, conforme comparecimento às unidades e aceite voluntário.

Para controle de potenciais fatores de confusão, utilizou-se regressão logística múltipla para estimar associações ajustadas entre as variáveis independentes e o desfecho.

### Tamanho do estudo

O tamanho amostral foi calculado a partir da média mensal de gestantes cadastradas nas quatro UBS, empregando-se a fórmula para amostras finitas, com prevalência estimada de toxoplasmose de 57,0% [10], nível de confiança de 95% (Z=1,96) e erro amostral de 5%. O cálculo resultou em uma amostra mínima de 185 gestantes, estratificada proporcionalmente à média de gestantes atendidas em cada UBS.

As participantes foram selecionadas por amostragem não probabilística, do tipo consecutiva, entre gestantes elegíveis em acompanhamento pré-natal nas UBS durante o período do estudo. Foram incluídas todas as gestantes elegíveis até o término da coleta de dados, respeitando a distribuição proporcional entre as unidades, ultrapassando o tamanho amostral mínimo previamente estimado.

### Métodos estatísticos

A base de dados foi construída no Microsoft Excel e analisada no pacote estatístico R (versão 4.1.0). Realizou-se análise descritiva, com medidas de tendência central e dispersão (média e desvio padrão, DP) para variáveis quantitativas e frequências absolutas e relativas para variáveis qualitativas.

As associações entre as variáveis independentes e o desfecho foram avaliadas por regressão logística simples, com estimativa das razões de chances (*odds ratio*, OR), intervalos de confiança de 95% (IC95%) e respectivos valores de p pelo teste de Wald. Variáveis com p-valor<0,200 na análise bivariada foram incluídas no modelo de regressão logística múltipla. Considerando o número de variáveis investigadas e o objetivo exploratório do estudo, utilizou-se o método de eliminação regressiva para seleção das variáveis no modelo final, iniciando-se com todas as variáveis elegíveis e removendo-se progressivamente aquelas sem significância estatística. No modelo final, foram estimadas OR ajustadas com IC95%. A colinearidade entre as variáveis independentes foi avaliada previamente à modelagem por meio da análise de correlação e da verificação de redundância conceitual entre variáveis que representavam dimensões semelhantes.

Em variáveis de múltipla resposta (por exemplo, origem dos alimentos), as frequências foram tratadas de forma independente. Adotou-se nível de significância de 5%. Variáveis quantitativas foram categorizadas em faixas para fins analíticos.

## Resultados

Participaram do estudo 240 gestantes; a idade média foi 26,8 anos (DP 6,84). A maioria autodeclarou-se branca (49,2%), vivia com companheiro (78,3%), tinha 9-11 anos de escolaridade (62,2%) e estava sem atividade remunerada no momento da entrevista (52,5%). Trabalho remunerado foi a fonte exclusiva de renda para 41,7%, e 17,5% dependiam do programa Bolsa Família. Em 37,9% dos domicílios o cônjuge foi apontado como principal responsável financeiro; 33,8% das participantes não possuíam fonte de renda própria. A maior frequência de renda mensal foi observada na faixa de um a dois salários mínimos (37,9%). O tempo médio de residência na região foi 9,9 anos (DP 7,8) (Tabela 1).

**Tabela 1:** Distribuição das características sociodemográficas, econômicas e relacionadas ao pré-natal das gestantes e intervalos de confiança de 95% (IC95%). Guarituba, Piraquara, Paraná, 2024 (n=240)

Variáveis	n (%)	IC95%
Faixa etária (anos)		
<18	10 (4,2)	2,3; 7,5
18-24	95 (39,6)	33,6; 45,9
25-29	50 (20,8)	16,2; 26,4
30-34	46 (19,2)	14,7; 24,6
35-39	30 (12,5)	8,9; 17,3
≥40	9 (3,7)	2,0; 7,0
Escolaridade (anos de estudo)		
1-4	8 (3,3)	1,7; 6,4
5-8	55 (22,9)	18,0; 28,6
9-11	154 (62,2)	57,9; 70,0
≥12	23 (9,6)	6,5; 14,0
Raça/cor da pele		
Branca	118 (49,2)	42,9; 55,4
Parda	101 (42,1)	36,0; 48,4
Preta	21 (8,7)	5,8; 13,0
Estado civil		
Casada	188 (78,3)	72,7; 83,1
Solteira	37 (15,4)	11,4; 20,5
Namoro	11 (4,6)	2,6; 8,0
Divorciada/viúva	4 (1,7)	0,6; 4,2
Responsável financeiro		
Cônjuge	91 (37,9)	32,0; 44,2
Compartilhado^a^	91 (37,9)	32,0; 44,2
Gestante	42 (17,5)	13,2; 22,8
Familiares	16 (6,7)	4,1; 10,6
Trabalho remunerado		
Não	126 (52,5)	46,2; 58,7
Sim	114 (47,5)	41,3; 53,8
Fonte de renda pessoal		
Trabalho	100 (41,7)	35,6; 48,0
Sem renda	81 (33,8)	28,1; 39,9
Benefício social	42 (17,5)	13,2; 22,8
Trabalho e benefício social	14 (5,8)	3,5; 9,6
Pensão	3 (1,3)	0,4; 3,6
Renda mensal da gestante (salário mínimo^b^)		
Sem renda	81 (33,8)	27,8; 39,7
<1	56 (23,3)	17,9; 28,7
1-2	91 (37,9)	31,8; 44,0
>2	12 (5,0)	2,3; 7,7
Tempo de moradia (anos)		
≤1	39 (16,3)	12,1; 21,4
>1-5	66 (27,5)	22,2; 33,5
>5-10	37 (15,4)	11,4; 20,5
>10	98 (40,8)	34,8; 47,1
Sorologia IgG anti-T. gondii		
Reagente	104 (43,3)	37,2; 49,7
Não reagente	136 (56,7)	50,3; 62,8
Tempo de gestação (semanas)		
≤13	32 (13,3)	9,6; 18,2
14-26	65 (27,1)	21,8; 33,0
≥27	143 (59,6)	53,3; 65,6
Início do pré-natal (trimestre)		
Primeiro	216 (90,0)	85,5; 93,2
Segundo	21 (8,8)	5,8; 13,0
Terceiro	3 (1,2)	0,4; 3,6
Número de consultas		
1-3	64 (26,7)	21,5; 32,6
4-6	76 (31,7)	26,1; 37,8
≥7	100 (41,6)	35,6; 48,0
Local pré-natal		
SUS	224 (93,3)	89,4; 95,9
Privado/convênio	1 (0,4)	0,1; 2,3
SUS e privado/convênio	15 (6,3)	3,8; 10,0

a Compartilhado: divisão entre cônjuge e/ou familiares. ^b^Salário mínimo vigente em 2024: R$1.412,00.

Foram identificadas 104 gestantes com IgG reagente, correspondendo à soroprevalência de 43,3% (IC95% 37,2; 49,7) (Tabela 1). Quatro gestantes apresentaram anticorpos IgM e IgG reagentes, todos com alta avidez de IgG, não sendo identificados casos compatíveis com infecção aguda durante a gestação.

Na análise bivariada, os fatores associados à maior chance de soropositividade foram faixa etária de 25-34 anos (OR 2,30; IC95% 1,08; 4,87) e ≥35 anos (OR 2,47; IC95% 1,39; 4,39); autodeclaradas pretas (OR 2,73; IC95% 1,05; 7,11); com quatro ou mais gestações (OR 3,97; IC95% 1,89; 8,31) (Tabela 2); e consumiam peixe proveniente de fontes alternativas ao comércio formal (OR 4,13; IC95% 1,76; 9,72) e salame proveniente de fontes alternativas (OR 4,58; IC95% 1,17; 18,00) (Tabela 3). As variáveis renda mensal da gestante ≥1 salário mínimo (OR 0,55; IC95% 0,30; 0,99) e não consumir peixe (OR 0,51; IC95% 0,28; 0,94) apresentaram efeito protetor (Tabelas 2 e 3).

No modelo ajustado, maiores chances de soropositividade foram observadas em mulheres de raça/cor da pele preta (OR 2,99; IC95% 1,07; 8,35), com quatro ou mais gestações (OR 2,04; IC95% 1,17; 4,14) e que consumiam peixe proveniente de fontes alternativas ao comércio formal (OR 1,62; IC95% 1,19; 2,20). As variáveis renda mensal ≥ 1 salário mínimo (OR 0,77; IC95% 0,62; 0,95) e o hábito de tratar a água destinada ao consumo (OR 0,42; IC95% 0,21; 0,81) apresentaram efeito protetivo (Tabela 4).

**Tabela 2:** Razão de chances (*odds ratio*, OR) e intervalos de confiança de 95% (IC95%) brutos das variáveis sociodemográficas, econômicas, relacionadas ao pré-natal e de convívio com gatos. Guarituba, Piraquara, Paraná, 2024 (n=240)

Variáveis	Total n (%)	Soropositivas n (%)	OR (IC95%)	p-valor
UBS				
Carlos Jess	105 (43,7)	45 (43,2)	1,00	-
Elfride Miguel de Oliveira	44 (18,3)	24 (23,1)	1,6 (0,79; 3,25)	0,193
Maria Francelina dos Santos	58 (24,2)	22 (21,2)	0,81 (0,42; 1,57)	0,541
Wanda Mallmann	33 (13,8)	13 (12,5)	0,87 (0,39; 1,93)	0,725
Faixa etária (anos)				
<25	105 (43,8)	33 (31,7)	1,00	-
25-34	96 (40,0)	51 (49,0)	2,30 (1,08; 4,87)	0,030
≥35	39 (16,2)	20 (19,2)	2,47 (1,39; 4,39)	0,002
Raça/cor da pele				
Branca	118 (49,2)	44 (42,3)	1,00	-
Parda	101 (42,1)	47 (45,2)	1,46 (0,85; 2,51)	0,167
Preta	21 (8,7)	13 (12,5)	2,73 (1,05; 7,11)	0,039
Convive com companheiro				
Não	52 (21,7)	17 (16,3)	1,00	-
Sim	188 (78,3)	87 (83,7)	1,77 (0,93; 3,39)	0,082
Anos de estudo				
1-8	64 (26,7)	33 (31,7)	1,00	-
9-11	153 (63,8)	64 (61,5)	0,68 (0,38; 1,21)	0,190
≥12	23 (9,6)	7 (6,7)	0,41 (0,15; 1,13)	0,086
Tempo de moradia (anos)				
≤5	105 (43,8)	46 (44,2)	1,00	-
>5-10	37 (15,1)	17 (16,4)	0,67 (0,30; 1,52)	0,342
>10	98 (40,8)	41 (39,4)	0,95 (0,55; 1,66)	0,865
Gestante como responsável financeiro				
Não	107 (44,6)	48 (46,2)	1,00	-
Sim	133 (55,4)	56 (53,8)	0,89 (0,53; 1,49)	0,669
Renda mensal da gestante (salário mínimo^a^)				
Sem renda	81 (33,8)	41 (39,4)	1,00	-
<1	56 (23,3)	26 (25,0)	0,85 (0,43; 1,67)	0,630
≥1	103 (42,9)	37 (35,6)	0,55 (0,30; 0,99)	0,046
Número de gestações				
1	84 (35,0)	26 (25,0)	1,00	-
2	69 (28,8)	29 (27,9)	1,62 (0,83; 3,15)	0,157
3	37 (15,4)	17 (16,3)	1,90 (0,86; 4,20)	0,115
≥4	50 (20,8)	32 (30,8)	3,97 (1,89; 8,31)	<0,001
Histórico de abortamento				
Não	181 (75,4)	72 (69,2)	1,00	
Sim	59 (24,6)	32 (30,8)	1,79 (0,99; 3,24)	0,053
Convívio com gatos (últimos 10 anos)				
Não	146 (60,8)	58 (55,8)	1,00	-
Sim	94 (39,2)	46 (44,2)	1,45 (0,86; 2,45)	0,161

a Salário mínimo vigente em 2024: R$1.412,00.

**Tabela 3:** Razão de chances (*odds ratio, OR)* e intervalos de confiança de 95% (IC95%) brutos das características de hábitos alimentares. Guarituba, Piraquara, Paraná, 2024 (n=240)

Variáveis	Total n (%)	Soropositivas n (%)	OR (IC95%)	p-valor
Consumo de peixe				
Sim	177 (73,7)	84 (80,8)	1,00	-
Não	63 (26,3)	20 (19,2)	0,51 (0,28; 0,94)	0,031
Origem do peixe				
Não consome	63 (26,3)	20 (19,2)	1,00	-
Comércio formal	139 (57,9)	59 (56,7)	1,59 (0,85; 2,97)	0,150
Fontes alternativas	38 (15,8)	25 (24,0)	4,13 (1,76; 9,72)	0,001
Consumo de carne bovina				
Sim	227 (94,6)	98 (94,2)	1,00	-
Não	13 (5,4)	6 (5,8)	1,13 (0,37; 3,46)	0,833
Origem da carne bovina				
Não consome	13 (5,4)	6 (5,8)	1,00	-
Comércio formal	220 (91,7)	92 (88,5)	0,84 (0,27; 2,58)	0,759
Fontes alternativas	7 (2,9)	6 (5,8)	7,00 (0,65; 7,73)	0,109
Consumo de carne de frango				
Sim	229 (95,4)	99 (95,2)	1,00	-
Não	11 (4,6)	5 (4,8)	1,09 (0,32; 3,69)	0,884
Origem da carne de frango				
Não consome	11 (4,6)	5 (4,8)	1,00	-
Comércio formal	209 (87,1)	85 (81,7)	0,82 (0,24; 2,78)	0,753
Fontes alternativas	20 (8,3)	14 (13,5)	2,80 (0,61; 12,86)	0,186
Consumo de carne suína				
Sim	185 (77,1)	78 (75,0)	1,00	-
Não	55 (22,9)	26 (25,0)	1,23 (0,67; 2,25)	0,502
Origem da carne suína				
Não consome	55 (22,9)	26 (25,0)	1,00	-
Comércio formal	171 (71,3)	68 (65,4)	0,74 (0,40; 1,36)	0,327
Fontes alternativas	14 (5,8)	10 (9,6)	2,79 (0,78; 9,98)	0,115
Consumo de salame				
Sim	164 (68,3)	72 (69,2)	1,00	-
Não	76 (31,7)	32 (30,8)	0,93 (0,54; 1,61)	0,794
Origem do salame				
Não consome	76 (31,7)	32 (30,8)	1,00	-
Comércio formal	151 (62,9)	62 (59,6)	0,96 (0,55; 1,68)	0,880
Fontes alternativas	13 (5,4)	10 (9,6)	4,58 (1,17; 18,00)	0,029
Consumo de linguiça				
Sim	34 (14,2)	13 (12,5)	1,00	-
Não	206 (85,8)	91 (87,5)	0,78 (0,37; 1,65)	0,517
Origem da linguiça				
Não consome	34 (14,2)	13 (12,5)	1,00	-
Comércio formal	201 (83,8)	88 (84,6)	1,26 (0,60; 2,65)	0,546
Fontes alternativas	5 (2,1)	3 (2,9)	2,42 (0,36; 16,5)	0,366
Consumo de leite				
Sim	224 (93,3)	95 (91,3)	1,00	-
Não	16 (6,7)	9 (8,7)	1,75 (0,63; 4,85)	0,280
Origem do leite				
Não consome	16 (6,7)	9 (8,7)	1,00	-
Comércio formal	211 (87,9)	86 (82,7)	0,54 (0,19; 1,49)	0,232
Fontes alternativas	13 (5,4)	9 (8,7)	1,75 (0,38; 8,14)	0,476
Consumo de queijo				
Sim	212 (88,3)	92 (88,5)	1,00	-
Não	28 (11,7)	12 (11,5)	0,98 (0,44; 2,17)	0,957
Origem do queijo				
Não consome	28 (11,7)	12 (11,5)	1,00	-
Comércio formal	177 (73,8)	74 (71,2)	0,96 (0,43; 2,14)	0,917
Fontes alternativas	35 (14,5)	18 (17,3)	1,41 (0,52; 3,84)	0,499
Consumo de carne crua ou malcozida				
Não	122 (50,8)	51 (49,0)	1,00	-
Sim	118 (49,2)	53 (51,0)	1,14 (0,68; 1,89)	0,627
Consumo de alimentos fora do domicílio				
Não	41 (17,1)	15 (14,4)	1,00	-
Sim	199 (82,9)	89 (85,6)	1,40 (0,70; 2,81)	0,340
Cultivo de horta no domicílio				
Não	183 (76,3)	80 (76,9)	1,00	-
Sim	57 (23,7)	24 (23,1)	0,94 (0,51; 1,71)	0,830
Higienização correta de hortifrútis				
Não	188 (78,3)	85 (81,7)	1,00	-
Não consome	6 (2,5)	1 (1,0)	0,24 (0,03; 2,11)	0,200
Sim	46 (19,2)	18 (17,3)	0,78 (0,40; 1,50)	0,457
Água exclusivamente da rede de abastecimento				
Não	144 (60,0)	64 (61,5)	1,00	-
Sim	96 (40,0)	40 (38,5)	0,89 (0,53; 1,51)	0,671
Tratamento da água para consumo				
Não	172 (71,7)	81 (77,9)	1,00	-
Sim	68 (28,3)	23 (22,1)	0,57 (0,32; 1,03)	0,063

**Tabela 4:** Razão de chances ajustada (*odds ratio*, OR) e respectivos intervalos de confiança de 95% (IC95%) dos fatores associados à presença de anticorpos IgG anti-*Toxoplasma gondii* segundo variáveis do estudo. Guarituba, Piraquara, Paraná, 2024 (n=240)

Categoria	OR ajustada (IC95%)	p-valor
Raça/cor da pele		
Branca/Parda	1,00	-
Preta	2,99 (1,07; 8,35)	0,037
Número de gestações		
1-3	1,00	-
≥4	2,04 (1,17; 4,14)	0,049
Renda mensal da gestante (salário mínimo)^a^		
<1	1,00	-
≥1	0,77 (0,62; 0,95)	0,017
Origem do peixe		
Não consome/comércio formal	1,00	-
Fontes alternativas	1,62 (1,19; 2,20)	0,002
Tratamento da água para consumo		
Não	1,00	-
Sim	0,42 (0,21; 0,81)	0,009

a Salário mínimo vigente em 2024: R$1.412,00.

## Discussão

A soroprevalência de anticorpos IgG anti-*Toxoplasma gondii* observada neste estudo indica exposição prévia relevante ao parasito na população investigada. Isso reflete condições ambientais e socioeconômicas que favorecem a manutenção do ciclo de transmissão no território. A associação da soropositividade com fatores sociodemográficos, reprodutivos e alimentares evidencia o caráter multifatorial da infecção e reforça a necessidade de estratégias integradas de vigilância e educação em saúde direcionadas a populações em contextos de vulnerabilidade.

Entre as limitações do estudo, destaca-se o delineamento transversal, que impede estabelecer relações causais entre exposições e desfecho e não permite avaliar desfechos neonatais ou confirmar infecção congênita. Também devem ser considerados possíveis vieses de memória e desejabilidade social em variáveis autorreferidas, além de potencial confundimento residual. A inclusão de gestantes vinculadas às UBS pode restringir a representatividade de mulheres sem acompanhamento pré-natal regular. Adicionalmente, a amplitude de alguns intervalos de confiança sugere menor precisão das estimativas em subgrupos com menor frequência. Os resultados sorológicos foram obtidos a partir dos registros das cadernetas de pré-natal e podem estar sujeitos a inconsistências decorrentes de erros de transcrição e ausência de padronização entre laboratórios, o que pode ter influenciado as estimativas observadas. Por outro lado, a padronização da coleta de dados e a completude das informações conferem consistência às análises realizadas.

A maior chance de soropositividade em gestantes com idade ≥25 anos provavelmente reflete exposição cumulativa ao parasito ao longo da vida [14]. Observou-se maior soropositividade entre gestantes autodeclaradas pretas. Entre aquelas com menor escolarização também se verificou maior frequência de soropositividade, embora sem associação estatisticamente significativa. Em conjunto, esses resultados sugerem a influência de desigualdades sociais e estruturais na exposição à infecção [15,16]. Mulheres em contextos socioeconômicos mais vulneráveis tendem a enfrentar barreiras no acesso a informações, serviços de saúde e condições adequadas de saneamento e alimentação segura, fatores que podem aumentar a exposição a riscos evitáveis [17-20]. 

A renda apresentou associação inversa com a soropositividade, sugerindo efeito protetor em quem possui maior renda mensal. Esse resultado é consistente com evidências de que melhores condições socioeconômicas favorecem práticas alimentares e de higiene mais seguras, além de maior acesso a serviços de saúde e informações sobre prevenção da toxoplasmose [10,14].

A soroprevalência de 43% observada neste estudo é compatível com valores descritos em regiões com contextos socioeconômicos semelhantes, indicando ampla exposição prévia ao parasito. Ao mesmo tempo, a presença de parcela expressiva de gestantes suscetíveis à infecção primária reforça a importância do acompanhamento sorológico durante o pré-natal para prevenção da toxoplasmose congênita [18,21-22].

Quatro gestantes apresentaram positividade para IgM e IgG, porém com alta avidez de IgG, sugerindo infecção anterior à gestação. O achado reforça a utilidade do teste de avidez para reduzir interpretações equivocadas no pré-natal. Ainda que incomuns, reativações da infecção em gestantes imunocomprometidas e casos de reinfecção podem resultar em transmissão congênita, reforçando a necessidade de vigilância sorológica adequada [4]. A maior soropositividade entre gestantes com quatro ou mais gestações pode estar associada à maior idade e ao aumento da exposição cumulativa ao parasito [14,18,22].

Não foi identificada associação entre convivência com gatos e soropositividade. Tal resultado reforça que a presença de felinos no domicílio não representa uma via direta de infecção humana, sendo a transmissão mais frequentemente associada à contaminação ambiental por oocistos presentes no solo, na água ou em alimentos [10,13,19,20,24-27].

Entre as exposições alimentares, destacou-se a associação com o consumo de peixe e salame provenientes de fontes alternativas ao comércio formal, o que sugere possível maior exposição associada a alimentos obtidos fora de cadeias com controle sanitário [19,23]. O tratamento da água apresentou efeito protetor no modelo ajustado. O dado corrobora o papel da qualidade da água na transmissão ambiental de *T. gondii*, uma vez que água e hortaliças contaminadas constituem vias relevantes de infecção [1,27].

No âmbito do Sistema Único de Saúde, a estimativa da soroprevalência de anticorpos anti-*T. gondii* em gestantes contribui para qualificar o planejamento das ações de atenção pré-natal e vigilância em saúde. A identificação de parcela expressiva de gestantes suscetíveis à infecção ressalta a importância da realização e do acompanhamento da sorologia durante a gestação, conforme preconizado nos protocolos assistenciais. Além disso, reconhecer fatores associados à soropositividade pode auxiliar no direcionamento de ações educativas voltadas à segurança da água e dos alimentos e na identificação de grupos que demandam maior acompanhamento durante o pré-natal. Esses achados também podem subsidiar o planejamento das ações de vigilância, a organização dos serviços e a alocação de recursos para a prevenção da toxoplasmose gestacional e congênita na atenção primária à saúde.

A soroprevalência de anticorpos IgG anti-*T. gondii* em gestantes atendidas na atenção primária da região do Guarituba foi de 43,3%, indicando exposição prévia expressiva nessa população. A soropositividade associou-se a características sociodemográficas e reprodutivas, bem como a práticas relacionadas à origem dos alimentos, enquanto melhores condições socioeconômicas e o tratamento da água destinada ao consumo apresentaram associação inversa. Esses achados reiteram a relevância de medidas de prevenção voltadas à segurança da água e dos alimentos e à redução de vulnerabilidades sociais em contextos atendidos pela atenção primária.

## Data Availability

Os dados estarão disponíveis mediante solicitação por *e-mail* ao autor correspondente.
