# Supplementary material for: Fatores associados à soroprevalência de infecção por Toxoplasma gondii em gestantes: estudo transversal, Piraquara, 2024
Source: Epidemiol Serv Saude. 2026 Oct 5;35:e20250708. [Article in Portuguese] doi: 10.1590/S2237-96222026v35e20250708.pt (PMC13647260; doi:10.1590/S2237-96222026v35e20250708.pt)
Supplement: Translation

# Translation: Factors associated with seroprevalence of *Toxoplasma gondii* infection in pregnant women: a cross-sectional study, Piraquara, Brazil, 2024

**DOI:** 10.1590/S2237-96222026v35e20250708.en

**Authors:** Fernanda Paula da Silva Torres, Nicole Muniz Ferreira Gonçalves, Vivien Midori Morikawa, Yanna Dantas Rattmann, Denise Siqueira de Carvalho, Marlene da Cruz Magalhães Buffon

**Affiliations:** 1 Universidade Federal do Paraná, Programa de Pós-Graduação em Saúde Coletiva, Curitiba, PR, Brazil; 2 Universidade Federal do Paraná, Programa de Residência Multiprofissional em Saúde da Família, Curitiba, PR, Brazil; 3 Universidade Federal do Paraná, Departamento de Saúde Coletiva, Curitiba, PR, Brazil

**Keywords:** Seroepidemiologic Studies, Pregnant Women, Toxoplasmosis, Toxoplasma gondii, Cross-Sectional Studies

## Abstract

**Objective:**

To estimate seroprevalence of *Toxoplasma gondii* infection in pregnant women in the Guarituba region of the municipality of Piraquara, Paraná, Brazil, and to analyze sociodemographic, clinical, and epidemiological factors associated with seropositivity.

**Methods:**

A cross-sectional study was conducted between March and September 2024 in four primary health care centers. Data were obtained through structured interviews and prenatal care records. Descriptive analysis and multiple logistic regression were performed to estimate odds ratios (OR) and 95% confidence intervals (95%CI).

**Results:**

240 pregnant women were included. Anti-*T. gondii* IgG antibody seroprevalence was 43.3% (95%CI 37.2; 49.7). Higher odds of seropositivity were observed in pregnant women of Black race/color (OR 2.99; 95%CI 1.07; 8.35), women who had had four or more pregnancies (OR 2.04; 95%CI 1.17; 4.14), and who consumed fish from sources other than formal commerce (OR 1.62; 95%CI 1.19; 2.20). Monthly income ≥1 minimum wage (OR 0.77; 95%CI 0.62; 0.95) and the habit of treating drinking water (OR 0.42; 95%CI 0.21; 0.81) showed inverse association.

**Conclusion:**

Seropositivity was associated with sociodemographic and dietary factors, while better socioeconomic conditions and treatment of drinking water showed inverse association.

## Introduction

Toxoplasmosis, an infection caused by the *Toxoplasma gondii* parasite, is a zoonosis with wide global distribution, having important implications for public health, especially in low- and middle-income countries [1]. It is estimated that around one-third of the world’s population is seropositive for *T. gondii*, with higher prevalence rates in regions of Latin America and South America [2]. Primary infection, as well as reactivation in immunosuppressed pregnant women or reinfection during pregnancy, can result in congenital toxoplasmosis, associated with serious outcomes such as spontaneous abortion, microcephaly, retinochoroiditis and cerebral calcifications [3,4].

Classified as a waterborne and foodborne disease, it can occur through ingestion of oocysts present in water or food contaminated with parasite sporulated oocysts, deposited in the environment through feces of infected felines, or through consumption of raw or undercooked meat containing tissue cysts [4].

In Brazil, notification of gestational toxoplasmosis became mandatory in 2016, and records have been progressively increasing. Between 2019 and 2023, the national averages were 33.0 cases per 10,000 pregnant women for gestational toxoplasmosis and 9.1 cases per 10,000 live births for the congenital form [5].

Piraquara is one of the 29 municipalities in the Metropolitan Region of Curitiba, Paraná. In this municipality, between 2019 and 2023, the average number of gestational toxoplasmosis cases was 104.2 per 10,000 pregnant women, while the average number of congenital toxoplasmosis cases was 29.4 per 10,000 live births. Both indicators were higher than the national averages [5,6]. Within the municipality, the Guarituba region accounted for 67% of reported cases in 2023, highlighting the unequal distribution of the disease [7]. In 2024, the region had approximately 51,000 inhabitants, corresponding to 43% of Piraquara’s total population. Recognized as an administrative region since 2016, Guarituba presents urban and environmental vulnerability, marked by unlawful land occupation and deficiencies in basic sanitation [6,8]. This scenario positions the region as a priority for public health actions. Investigating the serological profile of pregnant women in the region is relevant for gaining understanding about the proportion of women previously exposed and susceptible to infection.

From a Public Health perspective, understanding social and territorial inequalities is essential for addressing preventable diseases such as toxoplasmosis. The “One Health” approach complements this perspective by integrating human, animal and environmental dimensions, taking into account the interdependence between living conditions, environment and health in the control of zoonoses. The objective of this study was to estimate *T. gondii* infection seroprevalence in pregnant women from the Guarituba region and to analyze sociodemographic, clinical and epidemiological factors associated with seropositivity.

## Methods

### Study design and setting

This cross-sectional study was conducted between March and September 2024 in the Guarituba region, municipality of Piraquara, state of Paraná, Brazil. In 2024, the region had approximately 51,000 inhabitants, representing 42.9% of the population of Piraquara. The area is recognized as the largest unlawful land occupation in the state of Paraná and for its high social vulnerability.

The study took place in the four primary health care centers (*unidades básicas de saúde*, UBS) of the Guarituba Regional Health District. The proportional stratified sampling method took into consideration the average number of pregnant women receiving prenatal care at each health center: 135 at the Carlos Jess UBS; 86 at the Maria Francelina dos Santos UBS; 73 at the Elfride Miguel de Oliveira UBS; and 58 at the Wanda Mallmann UBS.

In the context of prenatal care in primary health care, serology testing for toxoplasmosis is part of the routine gestational examinations, as recommended by the Ministry of Health [9]. Serology testing for *Toxoplasma gondii* is requested at the beginning of gestational monitoring and, in susceptible pregnant women, should be repeated in each trimester of pregnancy.

### Participants

The study included pregnant women of any age, residing in the area covered by the primary health care centers in the Guarituba region, who were receiving prenatal care at one of the centers during the data collection period and who had undergone at least one routine prenatal examination with a record of serology testing for anti-*Toxoplasma gondii* antibodies.

The pregnant women were recruited regardless of their length of residence in the area.

Pregnant women under 18 years of age unaccompanied by a legal guardian, pregnant women without prenatal care records or without serology testing for *T. gondii*, as well as those who refused to participate in the study, were excluded.

Participants present at the health centers were invited after receiving explanations about the research and were included through consecutive sampling, based on their attendance at the centers during the study period and their voluntary acceptance.

### Variables

The primary outcome of the study was anti-*Toxoplasma gondii* IgG antibody seropositivity, classified as reactive or non-reactive according to the prenatal records. Presence of reactive IgG was considered indicative of previous exposure to the parasite. The laboratory variables IgM and IgG avidity, when available, were used only for serological characterization of the participants and identification of possible recent or previous infections prior to pregnancy. The variables analyzed were grouped into four domains:

a) age group (in years: <25; 25-34; ≥35), marital status, race/color, schooling (years of study: 1-8; 9-11; ≥12), work status, source of income, pregnant woman’s monthly income (no income; <1; ≥1 minimum wage), length of time resident in the area (in years: ≤5; >5-10; >10) and household and sanitation characteristics;
b) Obstetric health: gestational age at the time of the interview, trimester in which prenatal care started, number of consultations, number of pregnancies (1; 2; 3; ≥4); type of prenatal follow-up service and history of spontaneous abortions;
c) Living with cats: presence of felines in the home, type of food eaten by the cats, waste management, and the cats’ access to the outdoor environment;
d) Hygienic and food safety practices: consumption of animal products, consumption of raw or undercooked meat, fruit and vegetable cleaning, source of drinking water, and eating meals in places other than home.

### Data sources and measurement

The information was obtained through individual face-to-face interviews conducted in private rooms at the health centers, using a structured questionnaire based on literature on gestational toxoplasmosis, risk factors, lifestyle habits and socio-environmental conditions. The instrument contained 39 questions regarding sociodemographic characteristics, dietary and hygiene practices, obstetric history and contact with animals.

Serology test results (IgM, IgG and IgG avidity for *T. gondii*), classified as reactive or non-reactive, were obtained from the records in the pregnant women’s prenatal booklets, as noted by the physician responsible for prenatal care. The tests were performed according to the municipal health network prenatal care routine, by a laboratory linked to the public health system, using a methodology standardized by the service. The IgG avidity test, when recorded, was used to interpret cases with reactive IgM and IgG. High avidity was interpreted as infection prior to pregnancy, as recommended by the Ministry of Health [9].

The questionnaire was tested beforehand in a pilot study with pregnant women from the same population, but who were not included in the main sample, to assess clarity, language appropriateness and administration time. The adaptations resulting from the pilot test were incorporated into the final instrument. The interviews and data collection followed the same procedure in all the health centers.

### Bias

In order to reduce information bias, interviews were conducted individually in private rooms at the health centers, using language adapted to the participants’ level of understanding and administering a questionnaire tested beforehand in a pilot study. Laboratory information was obtained from records in the women’s prenatal booklets, as noted by the physician responsible for follow-up, using data generated in the prenatal care routine. It is possible that there may be recall bias and social desirability bias in self-reported variables related to eating and hygiene habits.

In order to minimize selection bias, eligible pregnant women receiving prenatal care at the four health centers in the Guarituba Regional Health District during the data collection period were included, recruited through consecutive sampling based on attendance at the health centers and voluntary acceptance.

In order to control for potential confounding factors, multiple logistic regression was used to estimate adjusted associations between the independent variables and the outcome.

### Study size

The sample size was calculated based on the monthly average of pregnant women registered at the four health centers, using the formula for finite samples, with an estimated toxoplasmosis prevalence rate of 57.0% [10], a 95% confidence level (Z=1.96) and a 5% sampling error. The calculation resulted in a minimum sample of 185 pregnant women, stratified proportionally to the average number of pregnant women cared for at each health center.

Participants were selected using non-probabilistic, consecutive sampling among eligible pregnant women receiving prenatal care at the health centers during the study period. All eligible pregnant women were included until the end of data collection, respecting proportional distribution between the health centers, thus exceeding the previously estimated minimum sample size.

### Statistical methods

The database was built using Microsoft Excel and analyzed using the R statistical package (version 4.1.0). Descriptive analysis was performed, with measures of central tendency and dispersion (mean and standard deviation, SD) for quantitative variables and absolute and relative frequencies for qualitative variables.

Associations between the independent variables and the outcome were assessed using simple logistic regression, with estimation of odds ratios (OR), 95% confidence intervals (95%CI), and respective p-values ​​using the Wald test. Variables with a p-value<0.200 in the bivariate analysis were included in the multiple logistic regression model. Considering the number of variables investigated and the exploratory objective of the study, the backward elimination method was used to select the variables in the final model, starting with all eligible variables and progressively removing those without statistical significance. In the final model, adjusted ORs with 95%CI were estimated. Collinearity between the independent variables was assessed prior to modeling through correlation analysis and verification of conceptual redundancy between variables representing similar dimensions.

In multiple-response variables (e.g., food origin), frequencies were treated independently. A 5% significance level was adopted. Quantitative variables were categorized into ranges for analytical purposes.

## Results

The study included 240 pregnant women. Their mean age was 26.8 years (SD 6.84). The majority self-identified as White (49.2%), lived with a partner (78.3%), had 9-11 years of schooling (62.2%), and were unemployed at the time of the interview (52.5%). Paid work was the sole source of income for 41.7%, and 17.5% depended on the *Bolsa Família* program (conditional cash transfer benefit). In 37.9% of households, the spouse was identified as the main money manager; 33.8% of participants had no source of income of their own. The highest frequency of monthly income was observed in the range of one to two minimum wages (37.9%). Average length of residence in the region was 9.9 years (SD 7.8) (Table 1).

**Table 1:** Distribution of sociodemographic, economic and prenatal-related characteristics of pregnant women and 95% confidence intervals (95%CI). Guarituba, Piraquara, Paraná, Brazil, 2024 (n=240)

Variables	n (%)	95%CI
Age group (years)		
<18	10 (4.2)	2.3; 7.5
18-24	95 (39.6)	33.6; 45.9
25-29	50 (20.8)	16.2; 26.4
30-34	46 (19.2)	14.7; 24.6
35-39	30 (12.5)	8.9; 17.3
≥40	9 (3.7)	2.0; 7.0
Schooling (years of study)		
1-4	8 (3.3)	1.7; 6.4
5-8	55 (22.9)	18.0; 28.6
9-11	154 (62.2)	57.9; 70.0
≥12	23 (9.6)	6.5; 14.0
Race/color		
White	118 (49.2)	42.9; 55.4
Mixed-race	101 (42.1)	36.0; 48.4
Black	21 (8.7)	5.8; 13.0
Marital status		
Married	188 (78.3)	72.7; 83.1
Single	37 (15.4)	11.4; 20.5
Dating	11 (4.6)	2.6; 8.0
Divorced/Widowed	4 (1.7)	0.6; 4.2
Money manager		
Spouse	91 (37.9)	32.0; 44.2
Shared^a^	91 (37.9)	32.0; 44.2
Pregnant woman	42 (17.5)	13.2; 22.8
Family members	16 (6.7)	4.1; 10.6
Paid work		
No	126 (52.5)	46.2; 58.7
Yes	114 (47.5)	41.3; 53.8
Source of personal income		
Work	100 (41.7)	35.6; 48.0
No income	81 (33.8)	28.1; 39.9
Social benefit	42 (17.5)	13.2; 22.8
Work and social benefit	14 (5.8)	3.5; 9.6
Pension	3 (1.3)	0.4; 3.6
Pregnant woman’s monthly income (minimum wage^b^)		
No income	81 (33.8)	27.8; 39.7
<1	56 (23.3)	17.9; 28.7
1-2	91 (37.9)	31.8; 44.0
>2	12 (5.0)	2.3; 7.7
Length of time living in neighborhood (years)		
≤1	39 (16.3)	12.1; 21.4
>1-5	66 (27.5)	22.2; 33.5
>5-10	37 (15.4)	11.4; 20.5
>10	98 (40.8)	34.8; 47.1
Anti-*T. gondii* IgG serology		
Reactive	104 (43.3)	37.2; 49.7
Non-reactive	136 (56.7)	50.3; 62.8
Length of pregnancy (weeks)		
≤13	32 (13.3)	9.6; 18.2
14-26	65 (27.1)	21.8; 33.0
≥27	143 (59.6)	53.3; 65.6
Start of prenatal care (trimester)		
First	216 (90.0)	85.5; 93.2
Second	21 (8.8)	5.8; 13.0
Third	3 (1.2)	0.4; 3.6
Number of consultations		
1-3	64 (26.7)	21.5; 32.6
4-6	76 (31.7)	26.1; 37.8
≥7	100 (41.6)	35.6; 48.0
Place of prenatal care		
SUS	224 (93.3)	89.4; 95.9
Private/outsourced	1 (0.4)	0.1; 2.3
SUS and private/outsourced	15 (6.3)	3.8; 10.0

a Shared: shared between spouse and/or family members. ^b^Minimum wage in force in 2024: R$1,412.00.

One hundred and four pregnant women were identified as having reactive IgG, corresponding to 43.3% seroprevalence (95%CI 37.2; 49.7) (Table 1). Four pregnant women presented reactive IgM and IgG antibodies, all with high IgG avidity; no cases compatible with acute infection during pregnancy were identified.

In the bivariate analysis, the factors associated with higher odds of seropositivity were being in the 25-34 age group (OR 2.30; 95%CI 1.08; 4.87) and the ≥35 age group (OR 2.47; 95%CI 1.39; 4.39); self-reporting race/color as Black (OR 2.73; 95%CI 1.05; 7.11); having had four or more pregnancies (OR 3.97; 95%CI 1.89; 8.31) (Table 2); and consuming fish from sources other than formal commerce (OR 4.13; 95%CI 1.76; 9.72) and salami from alternative sources (OR 4.58; 95%CI 1.17; 18.00) (Table 3). The pregnant woman’s monthly income ≥1 minimum wage variable (OR 0.55; 95%CI 0.30; 0.99) and the not consuming fish variable (OR 0.51; 95%CI 0.28; 0.94) showed a protective effect (Tables 2 and 3).

In the adjusted model, higher odds of seropositivity were observed in women of Black race/color (OR 2.99; 95%CI 1.07; 8.35), those who had had four or more pregnancies (OR 2.04; 95%CI 1.17; 4.14), and who consumed fish from sources other than formal commerce (OR 1.62; 95%CI 1.19; 2.20). The monthly income ≥ 1 minimum wage variable (OR 0.77; 95%CI 0.62; 0.95) and the habit of treating water intended for drinking variable (OR 0.42; 95%CI 0.21; 0.81) showed a protective effect (Table 4).

**Table 2:** Unadjusted odds ratios (OR) and 95% confidence intervals (95%CI) for sociodemographic, economic, prenatal and cat-related variables. Guarituba, Piraquara, Paraná, Brazil, 2024 (n=240)

Variables	Total n (%)	Seropositive n (%)	OR (95%CI)	p-value
Health Center				
Carlos Jess	105 (43.7)	45 (43.2)	1.00	-
Elfride Miguel de Oliveira	44 (18.3)	24 (23.1)	1.6 (0.79; 3.25)	0.193
Maria Francelina dos Santos	58 (24.2)	22 (21.2)	0.81 (0.42; 1.57)	0.541
Wanda Malmann	33 (13.8)	13 (12.5)	0.87 (0.39; 1.93)	0.725
Age group (years)				
<25	105 (43.8)	33 (31.7)	1.00	-
25-34	96 (40.0)	51 (49.0)	2.30 (1.08; 4.87)	0.030
≥35	39 (16.2)	20 (19.2)	2.47 (1.39; 4.39)	0.002
Race/color				
White	118 (49.2)	44 (42.3)	1.00	-
Mixed-race	101 (42.1)	47 (45.2)	1.46 (0.85; 2.51)	0.167
Black	21 (8.7)	13 (12.5)	2.73 (1.05; 7.11)	0.039
Living with a partner				
No	52 (21.7)	17 (16.3)	1.00	-
Yes	188 (78.3)	87 (83.7)	1.77 (0.93; 3.39)	0.082
Years of study				
1-8	64 (26.7)	33 (31.7)	1.00	-
9-11	153 (63.8)	64 (61.5)	0.68 (0.38; 1.21)	0.190
≥12	23 (9.6)	7 (6.7)	0.41 (0.15; 1.13)	0.086
Length of time living in neighborhood (years)				
≤5	105 (43.8)	46 (44.2)	1.00	-
>5-10	37 (15.1)	17 (16.4)	0.67 (0.30; 1.52)	0.342
>10	98 (40.8)	41 (39.4)	0.95 (0.55; 1.66)	0.865
Pregnant woman as money manager				
No	107 (44.6)	48 (46.2)	1.00	-
Yes	133 (55.4)	56 (53.8)	0.89 (0.53; 1.49)	0.669
Pregnant woman’s monthly income (minimum wage^a^)				
No income	81 (33.8)	41 (39.4)	1.00	-
<1	56 (23.3)	26 (25.0)	0.85 (0.43; 1.67)	0.630
≥1	103 (42.9)	37 (35.6)	0.55 (0.30; 0.99)	0.046
Number of pregnancies				
1	84 (35.0)	26 (25.0)	1.00	-
2	69 (28.8)	29 (27.9)	1.62 (0.83; 3.15)	0.157
3	37 (15.4)	17 (16.3)	1.90 (0.86; 4.20)	0.115
≥4	50 (20.8)	32 (30.8)	3.97 (1.89; 8.31)	<0.001
History of spontaneous abortion				
No	181 (75.4)	72 (69.2)	1.00	
Yes	59 (24.6)	32 (30.8)	1.79 (0.99; 3.24)	0.053
Living with cats (last 10 years)				
No	146 (60.8)	58 (55.8)	1.00	-
Yes	94 (39.2)	46 (44.2)	1.45 (0.86; 2.45)	0.161

a Minimum wage in force in 2024: R$1,412.00.

**Table 3:** Unadjusted odds ratios (OR) and 95% confidence intervals (95%CI) for dietary habit characteristics. Guarituba, Piraquara, Paraná, Brazil, 2024 (n=240)

Variable	Total n (%)	Seropositive n (%)	OR (95%CI)	p-value
Fish consumption				
Yes	177 (73.7)	84 (80.8)	1.00	-
No	63 (26.3)	20 (19.2)	0.51 (0.28; 0.94)	0.031
Origin of fish				
Does not consume	63 (26.3)	20 (19.2)	1.00	-
Formal commerce	139 (57.9)	59 (56.7)	1.59 (0.85; 2.97)	0.150
Alternative sources	38 (15.8)	25 (24.0)	4.13 (1.76; 9.72)	0.001
Beef consumption				
Yes	227 (94.6)	98 (94.2)	1.00	-
No	13 (5.4)	6 (5.8)	1.13 (0.37; 3.46)	0.833
Origin of beef				
Does not consume	13 (5.4)	6 (5.8)	1.00	-
Formal commerce	220 (91.7)	92 (88.5)	0.84 (0.27; 2.58)	0.759
Alternative sources	7 (2.9)	6 (5.8)	7.00 (0.65; 7.73)	0.109
Chicken consumption				
Yes	229 (95.4)	99 (95.2)	1.00	-
No	11 (4.6)	5 (4.8)	1.09 (0.32; 3.69)	0.884
Origin of chicken				
Does not consume	11 (4.6)	5 (4.8)	1.00	-
Formal commerce	209 (87.1)	85 (81.7)	0.82 (0.24; 2.78)	0.753
Alternative sources	20 (8.3)	14 (13.5)	2.80 (0.61; 12.86)	0.186
Pork consumption				
Yes	185 (77.1)	78 (75.0)	1.00	-
No	55 (22.9)	26 (25.0)	1.23 (0.67; 2.25)	0.502
Origin of pork				
Does not consume	55 (22.9)	26 (25.0)	1.00	-
Formal commerce	171 (71.3)	68 (65.4)	0.74 (0.40; 1.36)	0.327
Alternative sources	14 (5.8)	10 (9.6)	2.79 (0.78; 9.98)	0.115
Salami consumption				
Yes	164 (68.3)	72 (69.2)	1.00	-
No	76 (31.7)	32 (30.8)	0.93 (0.54; 1.61)	0.794
Origin of salami				
Does not consume	76 (31.7)	32 (30.8)	1.00	-
Formal commerce	151 (62.9)	62 (59.6)	0.96 (0.55; 1.68)	0.880
Alternative sources	13 (5.4)	10 (9.6)	4.58 (1.17; 18.00)	0.029
Sausage consumption				
Yes	34 (14.2)	13 (12.5)	1.00	-
No	206 (85.8)	91 (87.5)	0.78 (0.37; 1.65)	0.517
Origin of sausage				
Does not consume	34 (14.2)	13 (12.5)	1.00	-
Formal commerce	201 (83.8)	88 (84.6)	1.26 (0.60; 2.65)	0.546
Alternative sources	5 (2.1)	3 (2.9)	2.42 (0.36; 16.5)	0.366
Milk consumption				
Yes	224 (93.3)	95 (91.3)	1.00	-
No	16 (6.7)	9 (8.7)	1.75 (0.63; 4.85)	0.280
Origin of milk				
Does not consume	16 (6.7)	9 (8.7)	1.00	-
Formal commerce	211 (87.9)	86 (82.7)	0.54 (0.19; 1.49)	0.232
Alternative sources	13 (5.4)	9 (8.7)	1.75 (0.38; 8.14)	0.476
Cheese consumption				
Yes	212 (88.3)	92 (88.5)	1.00	-
No	28 (11.7)	12 (11.5)	0.98 (0.44; 2.17)	0.957
Origin of cheese				
Does not consume	28 (11.7)	12 (11.5)	1.00	-
Formal commerce	177 (73.8)	74 (71.2)	0.96 (0.43; 2.14)	0.917
Alternative sources	35 (14.5)	18 (17.3)	1.41 (0.52; 3.84)	0.499
Raw or undercooked meat consumption				
No	122 (50.8)	51 (49.0)	1.00	-
Yes	118 (49.2)	53 (51.0)	1.14 (0.68; 1.89)	0.627
Consumption of food outside of household				
No	41 (17.1)	15 (14.4)	1.00	-
Yes	199 (82.9)	89 (85.6)	1.40 (0.70; 2.81)	0.340
Vegetable garden at home				
No	183 (76.3)	80 (76.9)	1.00	-
Yes	57 (23.7)	24 (23.1)	0.94 (0.51; 1.71)	0.830
Correct cleaning of fruit and vegetables				
No	188 (78.3)	85 (81.7)	1.00	-
Does not consume	6 (2.5)	1 (1.0)	0.24 (0.03; 2.11)	0.200
Yes	46 (19.2)	18 (17.3)	0.78 (0.40; 1.50)	0.457
Water exclusively from mains supply				
No	144 (60.0)	64 (61.5)	1.00	-
Yes	96 (40.0)	40 (38.5)	0.89 (0.53; 1.51)	0.671
Treatment of drinking water				
No	172 (71.7)	81 (77.9)	1.00	-
Yes	68 (28.3)	23 (22.1)	0.57 (0.32; 1.03)	0.063

**Table 4:** Adjusted odds ratio (OR) and respective 95% confidence intervals (95%CI) of factors associated with the presence of anti-*Toxoplasma gondii* IgG antibodies according to study variables. Guarituba, Piraquara, Paraná, Brazil, 2024 (n=240)

Category	Adjusted OR (95%CI)	p-value
Race/color		
White/Mixed-race	1.00	-
Black	2.99 (1.07; 8.35)	0.037
Number of pregnancies		
1-3	1.00	-
≥4	2.04 (1.17; 4.14)	0.049
Pregnant woman’s monthly income (minimum wage)^a^		
<1	1.00	-
≥1	0.77 (0.62; 0.95)	0.017
Origin of fish		
Does not consume/formal commerce	1.00	-
Alternative sources	1.62 (1.19; 2.20)	0.002
Treatment of drinking water		
No	1.00	-
Yes	0.42 (0.21; 0.81)	0.009

a Minimum wage in force in 2024: R$1,412.00.

## Discussion

The anti-*Toxoplasma gondii* IgG antibody seroprevalence observed in this study indicates relevant prior exposure to the parasite in the population investigated. This reflects environmental and socioeconomic conditions that favor the maintenance of the transmission cycle in the territory. The association of seropositivity with sociodemographic, reproductive and dietary factors highlights the multifactorial nature of the infection and emphasizes the need for integrated surveillance and health education strategies directed at populations in vulnerable contexts.

The study’s cross-sectional design stands out among its limitations, as this design prevents establishment of causal relationships between exposures and outcomes and does not enable assessment of neonatal outcomes or confirmation of congenital infection. Possible recall and social desirability biases in self-reported variables should also be considered, in addition to potential residual confounding. The inclusion in the study of pregnant women linked to primary health care centers may restrict the representativeness of women without regular prenatal care. Additionally, the width of some confidence intervals suggests lower precision of estimates in subgroups with lower frequency. Serology test results were obtained from prenatal care records and may be subject to inconsistencies due to transcription errors and lack of standardization between laboratories, which may have influenced the estimates observed. On the other hand, the standardization of data collection and the completeness of the information provide consistency to the analyses performed.

The greater odds of seropositivity among pregnant women aged ≥25 years probably reflects cumulative lifetime exposure to the parasite [14]. Higher seropositivity was observed among pregnant women who self-reported their race/color as Black. A higher frequency of seropositivity was also found among those with lower education levels, although without statistically significant association. Taken together, these results suggest the influence of social and structural inequalities on exposure to infection [15,16]. Women in more vulnerable socioeconomic contexts tend to face barriers in accessing information, health services, adequate sanitation and safe food conditions, these being factors that can increase exposure to avoidable risks [17-20].

Income showed inverse association with seropositivity, suggesting a protective effect in those with higher monthly income. This result is consistent with evidence that better socioeconomic conditions favor safer food and hygiene practices, as well as greater access to health services and information on toxoplasmosis prevention [10,14].

The 43% seroprevalence rate observed in this study is consistent with values ​​described in regions with similar socioeconomic contexts, indicating widespread prior exposure to the parasite. At the same time, the presence of a considerable proportion of pregnant women susceptible to primary infection reinforces the importance of serological monitoring during prenatal care for preventing congenital toxoplasmosis [18,21-22].

Four pregnant women tested positive for IgM and IgG, but with high IgG avidity, suggesting infection prior to pregnancy. This finding reinforces the usefulness of the avidity test to reduce misinterpretations in prenatal care. Although uncommon, reactivation of infection in immunocompromised pregnant women and cases of reinfection can result in congenital transmission, emphasizing the need for adequate serological surveillance [4]. The higher seropositivity among pregnant women who had had four or more pregnancies may be associated with older age and increased cumulative exposure to the parasite [14,18,22].

No association was identified between living with cats and seropositivity. This result highlights that the presence of felines in the home does not represent a direct route of human infection, with transmission being more frequently associated with environmental contamination by oocysts present in soil, water or food [10,13,19,20,24-27].

Among food exposure, association with consumption of fish and salami from sources other than formal commerce stood out, suggesting possible higher exposure associated with foods obtained outside of supply chains with sanitary control [19,23]. Water treatment showed a protective effect in the adjusted model. This data corroborates the role of water quality in environmental transmission of *T. gondii*, since contaminated water and vegetables are relevant routes of infection [1,27].

Within the Brazilian Unified Health System (*Sistema Único de Saúde*, SUS), estimating anti-*T. gondii* antibody seroprevalence among pregnant women contributes to improving planning of prenatal care and health surveillance actions. Identifying a considerable proportion of pregnant women susceptible to infection highlights the importance of conducting and monitoring serology testing during pregnancy, as recommended in care protocols. Furthermore, recognizing factors associated with seropositivity can help guide educational actions focused on water and food safety and identify groups requiring closer monitoring during prenatal care. These findings can also support planning of surveillance actions, organization of services, and allocation of resources for prevention of gestational and congenital toxoplasmosis in primary health care.

Seroprevalence of anti-*T. gondii* IgG antibodies in pregnant women receiving primary care in the Guarituba region was 43.3%, indicating considerable prior exposure in this population. Seropositivity was associated with sociodemographic and reproductive characteristics, as well as practices related to food origin, while better socioeconomic conditions and treatment of drinking water showed inverse association. These findings reiterate the relevance of preventive measures aimed at water and food safety and the reduction of social vulnerabilities in contexts served by primary care.

## Data Availability

The data will be available upon request via email to the corresponding author.
